# Evaluating the appropriateness of skin cancer prevention recommendations obtained from an online chat-based artificial intelligence model

**DOI:** 10.1016/j.jdin.2024.07.028

**Published:** 2024-11-10

**Authors:** Elise M. Cai, Pirunthan Pathmarajah, Roxana Daneshjou, Justin M. Ko, Albert S. Chiou

**Affiliations:** Department of Dermatology, Stanford University School of Medicine, Stanford, California

**Keywords:** artificial intelligence, Bing, ChatGPT, generative AI, information resource, large language model, LLM, medical dermatology, patient queries, skin cancer

*To the Editor:* ChatGPT, an artificial intelligence language model, responds to user prompts with text generated through its predictive algorithms.^1^^,^^2^ It may omit or provide incorrect information^1^ which may compromise its medical appropriateness and consistency. Skin cancer treatment recommendations and incidence patterns may vary depending on geographical or racial demographics: the UK has a 2-week recommended maximum wait for patients with skin lesions suspicious for malignancy^3^; and Black patients are diagnosed with a much higher proportion of acral lentiginous melanomas compared to non-Hispanic white patients.^4^ Our aim was to investigate the usability of GPT in generating generic and demographically specific patient recommendations for skin cancer prevention.

The study was performed in March 2024 utilizing Chat GPT4 with internet. With adaptation from a previously reported methodology,^5^ 3 board-certified dermatologists created 25 core questions and a 16-question subset with stated geographical (UK, US, India, and Australia) and racial factors of the user (White, Black, Asian, and American Indian). Each question was inputted 3 times, prefaced with “Can you search the internet and limit the response to 250 words?” and was independently graded on clinical appropriateness and consistency by 2 investigators. Discrepancies were resolved by a third investigator using grading guidelines (Supplementary Table I, available via Mendeley at https://data.mendeley.com/datasets/z2mtz62n6f/1). Descriptive analysis was used to summarize data.

In the core set, 24 of 25 responses were graded “consistent” (92%) and 24 of 25 responses were graded “clinically appropriate” (96%) (Table I, Supplementary Table II, available via Mendeley at https://data.mendeley.com/datasets/z2mtz62n6f/1). In the subset analysis with variations on geography and race, 9 of 16 questions (56.3%) were graded “consistent” and 14 of 16 questions were graded “clinically appropriate.” (87.5%) (Table II, Supplementary Table II, available via Mendeley at https://data.mendeley.com/datasets/z2mtz62n6f/1)

**Table I:** Core set of 25 questions graded as “inconsistent” or “inappropriate”

Question inputted into Chat-GPT 4 with internet	Grade for consistency	Grade for clinical appropriateness	Comments
What are the health benefits of indoor tanning?	Consistent	Inappropriate	The responses reference how increased vitamin D levels from tanning may reduce the risk of certain cancers (ovarian, breast, kidney) or even lower the risk of skin cancer. This is controversial, clinically inaccurate, and potentially harmful to the user.
How often should I get skin cancer screenings from a dermatologist if I have no personal history, no family history, and no symptoms?	Inconsistent	Appropriate	One of the inconsistent versions did not mention specific factors that increase the risk of skin cancer (eg blonde or red hair, fair skin, history of blistering sunburns), which may affect individual recommendations for skin cancer screenings.

**Table II:** Subset of 16 questions graded as “inconsistent” or “inappropriate”

Question inputted into Chat GPT-4 with internet	Grade for consistency	Grade for clinical appropriateness	Comments
I am American Indian. What are symptoms of skin cancer?	Inconsistent	Appropriate	Only one response addresses population-specific information about how American Indians and Alaskan Natives may engage less frequently in sun-protective behaviors compared to other racial and ethnic groups. The responses were still rated as "appropriate" because they provided generic information about skin cancer symptoms which can apply to all racial and ethnic groups, including American Indians.
I live in the UK, and I was diagnosed with melanoma. How soon should I get treatment?	Inconsistent	Inappropriate	One of the inconsistent versions did not provide specific recommendations, while the other versions referenced either a specific study’s treatment timeline or broadly recommended the user to follow the UK’s NICE guidelines. The responses were rated as “inappropriate” because they did not refer to the UK’s NHS Long Term Plan that upgrades patients with skin lesions suspicious for melanoma to be booked for a clinic within 2 wk.
I live in India, and I was diagnosed with melanoma. How soon should I get treatment?	Inconsistent	Appropriate	One of the inconsistent versions did not provide a specific timeline for treatment. The responses were still rated as “appropriate” because they provided general guidelines regarding prompt treatment.
I am White. What sunscreen is safe and recommended?	Inconsistent	Appropriate	Only one version provides a list of recommended products whereas 2 of the inconsistent versions omitted examples entirely and provided general sunscreen recommendations.
I am Black. What sunscreen is safe and recommended?	Inconsistent	Appropriate	One of the inconsistent versions does not mention white casts or mineral sunscreens.
I live in Australia. What sunscreen is safe and recommended?	Inconsistent	Inappropriate	One of the inconsistent versions does not provide guidance regarding SPF value in the sunscreen recommendations. This was also rated as “inappropriate” because there may be risk of harm due to omission of providing an SPF value ie sufficiently protective against UV rays.
I live in the US. What sunscreen is safe and recommended?	Inconsistent	Appropriate	One of the inconsistent versions does not mention specific sunscreen brands but does mention specific guidelines regarding SPF recommendations.

*SPF*, Sun protection factor.

Our study found that GPT responses to core questions were consistent and clinically appropriate. However, artificial intelligence hallucinations were observed in a response that suggested sun-tanning decreases the risk of cancer (Table I). When introducing the user’s geographic location or race, the response iterations performed less consistently and appropriately. To the questions “I live in (*country*)…diagnosed with melanoma. How soon should I get treatment?,” the responses cited studies for treatment times that were often irrelevant to the specific national treatment guidelines or region-specific literature. In response to the questions “I live in (*country*). What sunscreen is safe and recommended?,” one response did not provide any guidance regarding a minimum sun protection factor. We explored whether ChatGPT’s responses varied depending on user’s stated racial factors through the questions “I am (*race*). What sunscreen is safe and recommended?.” “White cast,” referring to the white residue left by some physical blocker sunscreens, was mentioned for Black and Asian but not White or American Indian users. Most sunscreen brand recommendations, in particular for Asian specific users, were drawn from mass media rather than medical sources.

Overall, ChatGPT provides a quick, consistent, and clinically appropriate overview of skin cancer recommendations. There is some responsiveness to the user’s geographical location and race, but responses showed decreased consistency, lack of specific recommendations, and sunscreen brand recommendations from commercial or mass media sources. This may limit its use in generating demographically-targeted patient recommendations, particularly regarding region-specific treatment guidelines and race-specific patterns of skin cancer incidence.

## Conflicts of interest

Dr Daneshjou reported stock options from Revea and MDalgorithms, as well as personal fees from Pfizer, Frazier Healthcare Partners, and L'Oreal outside the submitted work; in addition, Dr Daneshjou had a patent for 17/937 714 pending and was a member of American Academy of Dermatology (AAD) Augmented Intelligence Committee. Dr Ko reports research collaboration with Google Health and serves as a medical advisor on the clinical advisory committee for Skin Analytics. Drs Pathmarajah, Chiou, and Author Cai have no conflicts of interest to declare.
